# LRPAP1 is released from activated microglia and inhibits microglial phagocytosis and amyloid beta aggregation

**DOI:** 10.3389/fimmu.2023.1286474

**Published:** 2023-11-16

**Authors:** Kyle M. Reid, Guy C. Brown

**Affiliations:** Department of Biochemistry, University of Cambridge, Cambridge, United Kingdom

**Keywords:** microglia, LRPAP1, receptor-associated protein, LRP1, LDL receptor, brain, amyloid beta, aggregation

## Abstract

Low-density lipoprotein receptor-related protein-associated protein 1 (LRPAP1), also known as receptor associated protein (RAP), is an endoplasmic reticulum (ER) chaperone and inhibitor of LDL receptor related protein 1 (LRP1) and related receptors. These receptors have dozens of physiological ligands and cell functions, but it is not known whether cells release LRPAP1 physiologically at levels that regulate these receptors and cell functions. We used mouse BV-2 and human CHME3 microglial cell lines, and found that microglia released nanomolar levels of LRPAP1 when inflammatory activated by lipopolysaccharide or when ER stressed by tunicamycin. LRPAP1 was found on the surface of live activated and non-activated microglia, and anti-LRPAP1 antibodies induced internalization. Addition of 10 nM LRPAP1 inhibited microglial phagocytosis of isolated synapses and cells, and the uptake of Aβ. LRPAP1 also inhibited Aβ aggregation *in vitro*. Thus, activated and stressed microglia release LRPAP1 levels that can inhibit phagocytosis, Aβ uptake and Aβ aggregation. We conclude that LRPAP1 release may regulate microglial functions and Aβ pathology, and more generally that extracellular LRPAP1 may be a physiological and pathological regulator of a wide range of cell functions.

## Highlights

- Stressed microglia release LRPAP1 at levels that inhibit microglial phagocytosis of synapses and cells, and reduce amyloid beta fibrillization and clearance.

## 1 Introduction

Low density lipoprotein (LDL) receptor related protein 1 (LRP1) is a plasma membrane receptor mediating endocytosis, phagocytosis, chemotaxis and signaling for extracellular ligands, including apolipoprotein E (ApoE), lipoproteins, α_2_-macroglobulin, proteases, extracellular matrix proteins, growth factors, complement factors, heat shock proteins and dozens of other natural ligands (1–5). LRP1 is part of the LDL receptor family, which includes LRP2 (megalin), LRP8 (ApoER2), the LDL receptor and the VLDL (very low density lipoprotein) receptor (6).

LDL receptor-related protein-associated protein 1 (LRPAP1), also known as receptor-associated protein (RAP), is a chaperone for LRP1 and related LDL receptors (7), binding these receptors in the endoplasmic reticulum (ER) and enabling translocation to the Golgi and plasma membrane without denaturation or binding of ligands (8). LRPAP1 antagonizes all known natural ligands of the LDL receptor family with nanomolar affinity (9–14). However, it is not known whether extracellular LRPAP1 is a physiological (or pathological) inhibitor/regulator of these receptors, because it is not known whether LRPAP1 is released extracellularly in physiological conditions and concentrations sufficient to inhibit the receptors. If this did occur, LRPAP1 could regulate the affinity of these receptors for all these ligands, and a wide range of cellular functions.

Microglia are resident macrophages of the central nervous system, and regulate brain development, physiology, inflammation and pathology (15). Microglia express LRP1, which regulates multiple microglial activities, including phagocytosis, inflammation and pathology (16, 17). Alzheimer’s disease is the main cause of dementia, and is driven in part by the aggregation of Aβ (18, 19). Microglial LRP1 mediates Aβ uptake and activation of microglia (20–22). Genetic variants of LRPAP1 have been linked to dementia, late-onset Alzheimer’s disease and Parkinson’s disease (23–25), and such variants may be unable to bind LRP1 correctly (26). LRP1 on endothelial cells of the blood-brain barrier mediates export of Aβ out of the brain (27), and neurons, astrocytes and microglia use LRP1 to uptake Aβ (28), so LRPAP1 could regulate the clearance of Aβ by all these cell types. LRPAP1 can bind Aβ (29), and could also regulate uptake as an extracellular chaperone.

Recently, we reported that activated and ER-stressed microglia can release the ER-resident chaperone calreticulin, which then activated microglia and acted as an extracellular chaperone for Aβ (30). This led us to investigate whether other ER-resident chaperones, such as LRPAP1, are released by activated microglia. Our aim here was to discover whether microglia release LRPAP1 in various conditions, and if so whether relevant LRPAP1 concentrations do anything to microglial physiology and pathology, in particular to microglial phagocytosis and Aβ aggregation.

## 2 Materials and methods

### 2.1 Materials

All cell culture reagents were from Invitrogen (Paisley, UK), unless otherwise indicated. Culture treatments were procured as follows: staurosporine (from *Streptomyces* sp.) was from Abcam (Cambridge, UK); peptide synthesized unlabeled and HiLyte™ Fluor 555 human amyloid-beta (1-42) was purchased from Anaspec (CA, USA); mouse anti-IgG1 isotype control was from BioLegend (CA, USA); mouse seroblock FcR BUF041A was from BioRad (CA, USA); recombinant human LRPAP1 protein was from Enzo Life Sciences (NY, USA); cytochalasin D, lipopolysaccharide (LPS; from *Salmonella Enterica* serotype typhimunium), polymyxin B sulfate salts and tunicamycin (from *Streptomyces* sp.) were from Sigma-Aldrich (MO, USA); antibodies to human and mouse LRPAP1 were from SinoBiological (Beijing, China); rabbit anti-IgG isotype control was from Southern Biotech (AL, USA); recombinant human tumour necrosis factor α and thioflavin T were from ThermoFisher (MA, USA).

### 2.2 Cell culture and treatments

The immortalized cell lines BV-2 (ECACC Cat# 0356, RRID: CVCL_0182) and CHME3 (ATCC Cat# CRL-3304, RRID: CVCL_II76) were maintained as previously described (31, 32). Neither cell line is listed as a commonly misidentified cell line by the International Cell Line Authentication Committee.

Cells were treated as follows: BUF041A was used at 5 µg/mL for 20 minutes. Cytochalasin D was used at 10 µM for 2 or 4 hours. LPS was used at 0.1 or 0.8 µg/mL 24 hours. LRPAP1 was used at 10 nM or 100 nM for 2 or 3 hours, 10 nM or 500 nM for 4 hours, and 500 nM for 24 hours. Monomeric Aβ was used at 500 nM for 4 hours, or 5 µM for 24 hours. Polymyxin B was used at 10 U/mL for 24 hours. Thioflavin T was used at 10 µM for 24 hours. TNF-α was used at 50 ng/mL for 24 hours. Tunicamycin was used at 2 µg/mL for 24 hours. Staurosporine at used at 1000nM for 24 hours.

### 2.3 Cell viability

Cell viability, defined as the percentage of non-necrotic cells, was measured at indicated endpoints by differential dye uptake of propidium iodide (identifying necrotic cells) and Hoechst 33342 (identifying all cells) using a fluorescent microscope (EVOS M5000). Alexa Fluor 488-tagged isolectin B4 was used to identify BV-2 microglia. For each technical replicate, the entire well was imaged, and the number of cells quantified using QuPath (version 0.4.3).

### 2.4 Measurement of LRPAP1 release

Measurement of protein levels from cell culture supernatants were conducted by SDS-PAGE western blot. BV-2 microglia were plated at 5 x 10^4^ cells/100 µL/well in serum-free culture medium and treated accordingly. CHME3 microglia were plated at 1.5 x 10^4^ cells/100µL/well in serum-free culture medium and treated accordingly. Conditioned media of treated cultures were transferred into eppendorfs and thereafter kept on ice when possible. To remove any debris from cell supernatants, eppendorfs were centrifuged (500g RCF, 5 minutes). To prepare samples for gel electrophoresis, the top fraction of supernatant from each eppendorf was removed and supplemented with 25% NuPAGE™ LDS sample buffer and 50mM dithiothreitol (DTT). To quantify densitometry values as protein amount, recombinant LRPAP1 was serially diluted to form a three-point standard curve, which were loaded alongside experimental samples (**Supplementary Figure 1**). Proteins were separated by SDS-PAGE, resolved on NuPAGE 4-12% Bis-Tris gels (Invitrogen), then transferred onto PVDF membranes using the iBlot® dry blotting system. Membranes were blocked in PBS (5% milk), then probed with primary antibodies against LRPAP1 (1:500) overnight at 4°C. Membranes were washed with TBST-Tween 20 and incubated with IRDye^®^ 800CW donkey anti-rabbit IgG secondary antibody (1:5000, Licor). Membranes were imaged using the LI-COR Odyssey® CL_X_ at 700nm and 800nm wavelengths. Band intensities were quantified using Image Studio™.

### 2.5 Immunocytochemistry

Immunocytochemistry was used to visualize the expression of molecules on the surface of microglia. BV-2 microglia were plated to a density of 5.0 x 10^4^ cells/100µL/well in serum-free culture medium and treated. CHME3 microglia were plated to a density of 1.5 x 10^4^ cells/100µL/well in serum-free culture medium and treated. Culture media was removed, and cultures were incubated in ice cold PBS containing antibodies to isotype IgG or LRPAP1 for 1 hour. Wells were washed with ice-cold PBS three times and replaced with PBS containing Alexa Fluor™ 488-tagged anti-rabbit IgG for 1 hour. Culture wells were removed of their supernatants, washed with ice-cold PBS three times, and imaged using the EVOS M5000 fluorescence microscope. To examine antibody internalization, cultures were incubated at room temperature for allotted periods of time. LRPAP1 surface expression was measured by flow cytometry using the Attune NxT flow cytometer (see section *2.10. Flow cytometry*).

### 2.6 Phagocytosis assays

BV-2 microglia were plated to a density of 2.5 x 10^4^ cells/100 µL/well in low-serum culture medium and incubated for 24 hours (37°C, 5% CO_2_). CHME3 microglia were plated to a density of 2 x 10^4^ cells/100 µL/well in low-serum culture medium and incubated for 24 hours (37°C, 5% CO_2_).

Isolated synaptosomes were prepared as described by Dunkley et al. (33). Synaptosomes were incubated with 10 µM pHrodo in CO_2_-infused HBK buffer (HEPES-buffered Krebs-like; 15 minutes, 37°C, dark). pHrodo-stained rat synaptosomes underwent three successive wash stages whereby the cells were centrifuged (20,000g RCF, 5 minutes), media was removed, and the pellet was resuspended in fresh HBK buffer. To induce apoptosis in PC12 pheochromocytomas, cells were irradiated with 36 J/cm^2^ of UV-A (365 nm) using the UVP Crosslinker CL-3000, then incubated for 3 hours (37°C, 5% CO_2_). Apoptotic PC12 cells were labelled with pHrodo following the same protocol used to label synaptosomes, except instead of HBK buffer, PBS was used. pHrodo-labelled synaptosomes or PC12 cells were added directly to treated microglial cultures. Synaptosome co-cultures were incubated for 3 hours (37°C, 5% CO_2_). PC12 co-cultures were incubated for 2 hours (37°C, 5% CO_2_). After incubations, wells were washed with ice cold PBS, then imaged using the EVOS M5000 fluorescence microscope. For co-cultures containing BV-2 microglia, well media was replaced with 2 µg/mL Alexa Fluor™ 488-tagged isolectin B4 (IB4) in PBS. Phagocytosis was measured by flow cytometry using the Attune NxT flow cytometer (see section *2.10. Flow cytometry*).

### 2.7 Preparation of monomeric amyloid beta

Lyophilized unlabeled or labelled amyloid beta (Aβ) peptide spanning N-terminal residues 1-42 (Aβ_1-42_) was solubilized in 1,1,1,3,3,3-hexafluoro-isopropanol (HFIP, Sigma) as previously described (34). After addition of HFIP to the lyophilized Aβ, the container was sealed and incubated at room temperature for 1 hour, then diluted in deionized water and further incubated for 20 minutes. The Aβ solution was centrifuged (12,000 RPM, 15 minutes) and the supernatant was transferred into an empty eppendorf, where the HFIP was evaporated off. For long-term storage, the Aβ solution was aliquoted into smaller volumes and dried to a clear film under a stream of nitrogen. For experimentation, Aβ was resolubilized in dimethyl sulfoxide (DMSO, Sigma), diluted in PBS, and sonicated for 10 minutes prior to use.

### 2.8 Measurement of Aβ uptake

BV-2 microglia were plated at 5 x 10^4^ cells/100 µL/well in low-serum culture medium and treated with or without LPS (100 ng/mL) for 24 hours. CHME3 microglia were plated at 1.5 x 10^4^ cells/100 µL/well in low-serum culture medium and treated with or without LPS (0.8 µg/mL) for 24 hours. Cultures were treated accordingly alongside HiLyte™ Fluor 555-labelled Aβ (500 nM), and uptake of Aβ by treated microglia was measured over 4 hours under incubating conditions (37°C, 5% CO_2_). Wells were washed with ice-cold PBS three times to remove soluble Aβ, and cultures were imaged using the EVOS M5000 fluorescence microscope (4X magnification; RFP channel). For co-cultures containing BV-2 microglia, well media was replaced with 2 µg/mL Alexa Fluor™ 488-tagged isolectin B4 (IB4) in PBS. Aβ uptake was measured by flow cytometry using the Attune NxT flow cytometer (see section *2.10. Flow cytometry*).

### 2.9 Measurement of Aβ fibrillization

Aβ fibrillization was measured by thioflavin T (Sigma) fluorescence as previously described (35). Briefly, unlabeled Aβ was diluted to a concentration of 5µM in PBS (10 µM Thioflavin T) ± 500 nM LRPAP1. Thioflavin T alone served as a background fluorescence control for fibrillization and was subtracted from all values at each fluorescence reading. Conditions were added to wells of a black 96-well microtiter plate with a flat, clear bottom. Wells were sealed to prevent evaporation, and the plate was placed into the FLUOstar OPTIMA microplate reader set to an incubating temperature of 31.5°C. Fluorescence readings (absorbance: 440 nm; emission: 480 nm) were taken every 10 minutes for 24 hours, with 10 seconds of orbital shaking preceding each measurement. At each experimental endpoint, wells were imaged for Thioflavin T fluorescence by fluorescence microscopy using the RFP channel of the EVOS M5000 (ThermoFisher). To measure both deposited and free-floating Aβ, Z-stack images were taken of the entire well, and maximum intensity projections were generated.

### 2.10 Flow cytometry

To analyze microglial surface expression, phagocytosis or Aβ uptake by flow cytometry, cultures were resuspended by enzymatic detachment with 0.05% trypsin in PBS (ice cold). To remove the trypsin, detached cells were centrifuged (100g RCF, 5 minutes), media was removed, and the cell pellet was resuspended in fresh PBS (ice cold). Suspensions were kept on ice until sampling by the Attune NxT flow cytometer. The rate of sample volume draw for cytometry was regulated to 500 µL/minute. Digital gating accounted for doublet discrimination and limited the analyses to the targets of interest. Mixed cell suspensions of similar cell sizes were avoided to prevent ambiguity when assigning event clouds to individual cell populations. Where possible, 10,000 events denoting the target of interest were acquired. Events were analyzed for forward-scatter (∝ event size), side-scatter (∝ event granularity), and fluorescence. Data analysis was performed using Attune NxT Cytometric Software (ThermoFisher).

Data acquired by flow cytometry was presented as either i) median fluorescence intensity (displayed as percentage uptake or surface expression to a control condition) or ii) percentage of phagocytosing cells, measured using the ‘fluorescence gate-shift’ method. The ‘fluorescence gate-shift’ method measures the percentage of phagocytosing cells by generating a gate around the event population of microglia treated with Cytochalasin D (which abolished phagocytosis (36)), so that 1% of total events are in the ‘fluorescence positive’ gate (i.e., positively gated events (cells) emit fluorescence positive for phagocytosis).

### 2.11 Statistical analysis

Statistical analyses were conducted using GraphPad Prism v10.0.1. Statistical differences between three or more groups were analyzed by one-way repeated measures or mixed model ANOVA followed by Dunnett’s or Šidák multiple comparisons *post-hoc* test. Where two independent variables were tested between two or more groups, datasets were analyzed by ordinary two-way ANOVA followed by Turkey multiple comparisons *post-hoc* test. Statistical differences between normality of acquired data was tested by Shapiro-Wilk test. Error bars represent the standard error of the mean of experiments (SEM). p-values indicate the reliability of the estimate and were considered significant when p<0.05. The null hypothesis was accepted when p≥0.05.

## 3 Results

**Figure 1:**
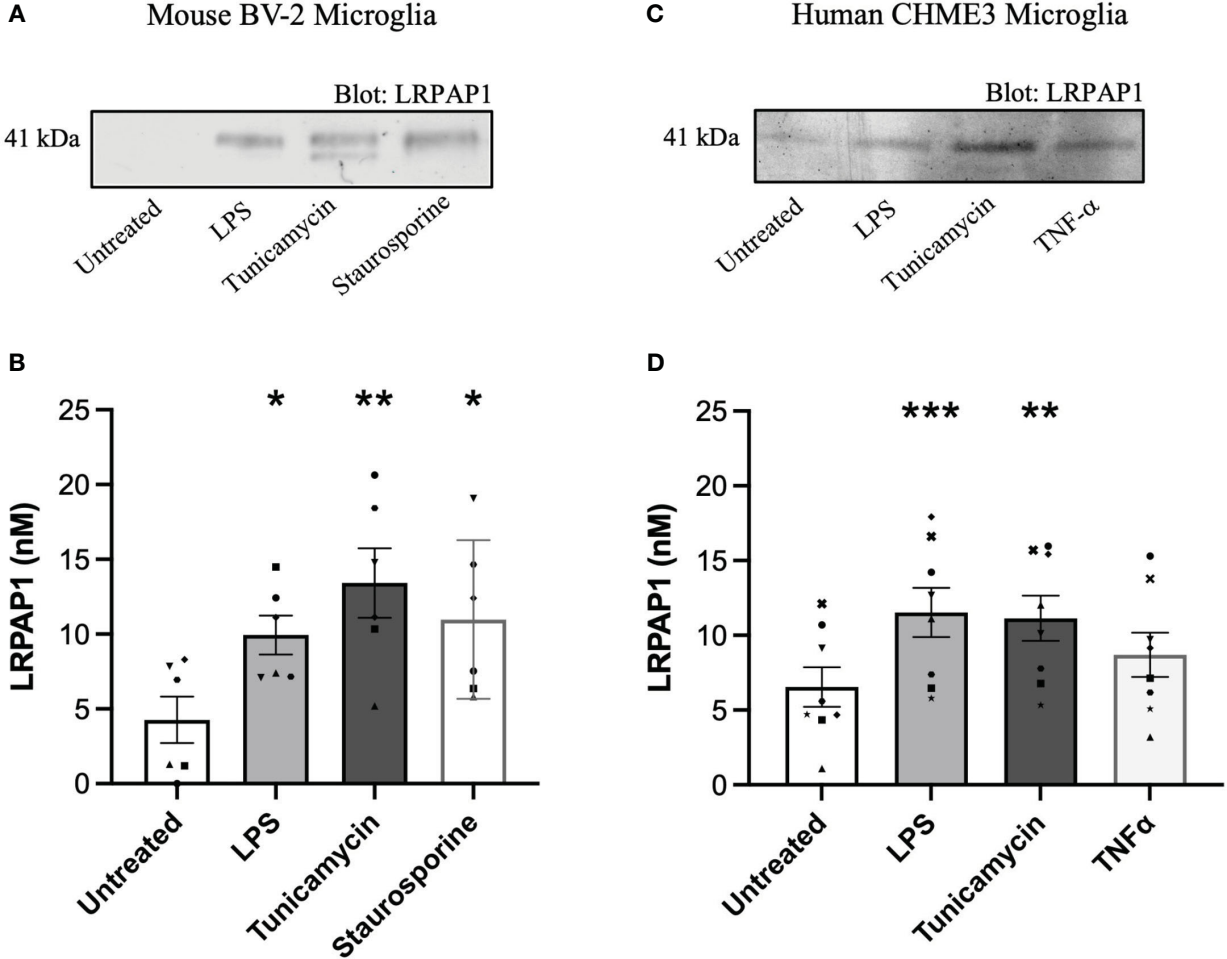
LRPAP1 is released from stressed microglia. Murine BV-2 microglia were treated with or without LPS (100 ng/mL), tunicamycin (2 µg/mL) or staurosporine (1000 nM) for 24 hours. Human CHME3 microglia were treated with or without LPS (0.8 µg/mL), tunicamycin (2 µg/mL), or TNF-α (50 ng/mL) for 24 hours. Supernatants were removed and assessed for LRPAP1 release by quantitative densitometry of western blots using internal three-point standard curves of recombinant LRPAP1. **(A)** Representative western blot image of LRPAP1 release from treated BV-2 microglia. **(B)** LRPAP1 release into the wells of treated BV-2 cultures, represented as nanomolar concentration. **(C)** Representative western blot image of LRPAP1 release from treated CHME3 microglia. **(D)** LRPAP1 release into the wells of treated CHME3 cultures, represented as nanomolar concentration. Data presented as LRPAP1 molarity values, with error bars representing the SEM of **(B)** 6 and **(D)** 8 independent experiments. Statistical comparisons were made to the untreated control, by one-way ANOVA with Dunnett’s *post-hoc* test. ns: p≥0.05, *p<0.05, **p<0.01, ***p<0.001. LPS, Lipopolysaccharide; LRPAP1, Low-density lipoprotein protein receptor-related protein-associated protein 1; TNF-α, Tumour necrosis factor α.

We wanted to test whether LRPAP1 was released by microglia in various stress conditions. To do this, we used the mouse microglial cell line BV-2 and the human microglial cell line CHME3 (also known as HMC3), and measured LRPAP1 concentrations in the cell culture medium after various treatments for 24 hours by western blots. Activation of BV-2 microglia with the TLR4 agonist lipopolysaccharide (LPS) resulted in a significant increase in extracellular LRPAP1, increasing about 2.5 fold to 10 nM LRPAP1 (**Figures 1A, B**). An inducer of ER stress, tunicamycin, also significantly increased extracellular LRPAP1, to about 12.5 nM LRPAP1 (**Figures 1A, B**). By inhibiting N-linked glycosylation, tunicamycin also induced the appearance of a lower-molecular-weight band in the LRPAP1 western blot, which may correspond to non-glycosylated LRPAP1 (**Figure 1A**). An inducer of apoptosis, staurosporine, also significantly increased extracellular LRPAP1 (**Figures 1A, B**). As previously reported by us, under these conditions, treatment with LPS and tunicamycin did not cause significant cell death, while staurosporine killed the cells (30).

In human CHME3 microglia, we measured LRPAP1 release in response to LPS, tunicamycin and TNF-α, and found LPS and tunicamycin induced significant LRPAP1 release to about 10 nM LRPAP1 (**Figures 1C, D**). Cell viability was unaffected by these treatments (**Supplementary Figure 2**). We did not include a loading control in each of our western blot studies because there is no such protein in the extracellular medium to standardize to, and because we are not interested in the ratio of one extracellular protein to another, but rather the absolute concentration of LRPAP1. Thus, inflammation (induced by LPS) and ER stress (induced by tunicamycin) appear to cause LRPAP1 release from mouse and human microglial cell lines, resulting in extracellular LRPAP1 levels of about 10 nM.

**Figure 2:**
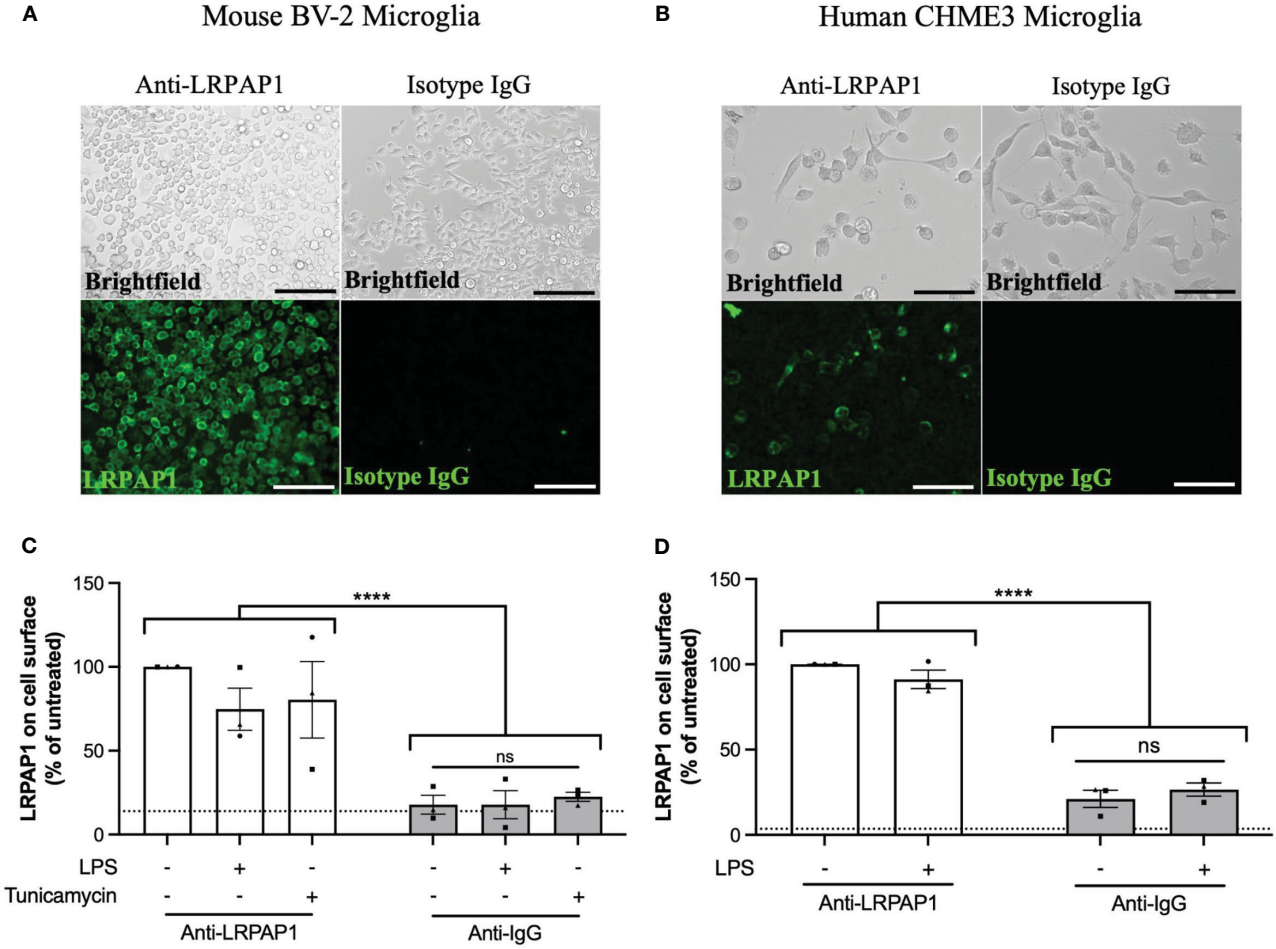
LRPAP1 is present on the surface of microglia. BV-2 microglia were plated at 5 x 10^4^ cells/100 µL/well in serum-free medium and treated with LPS (100 ng/mL) or tunicamycin (2 µg/mL) for 24 hours. CHME3 microglia were plated at 1.5 x 10^4^ cells/100 µL/well in serum-free medium and treatment with or without LPS (0.8 µg/mL) for 24 hours. **(A, B)** Representative fluorescence images of untreated **(A)** BV-2 microglia (Scale = 150 µm) or **(B)** CHME3 microglia (Scale = 150 µm) stained with antibodies against either anti-LRPAP1 or anti-IgG isotype control antibodies, followed by an Alexa Fluor 488-tagged anti-rabbit IgG secondary antibody. Cultures were kept at 4°C during the staining phase. **(C, D)** LRPAP1 on the surface of **(C)** BV-2 microglia and **(D)** CHME3 microglia, measured by flow cytometry using anti-LRPAP1 antibody, and expressed as median fluorescence intensity (MFI) as a % of the untreated control MFI. Unstained cultures served as the autofluorescence control and were subtracted from the final MFI values. Dotted line represents the percentage mean fluorescence of microglia stained with Alexa Fluor 488-tagged anti-rabbit IgG secondary antibodies (i.e. secondary control). Data is presented as percentage surface LRPAP1 expression values, with error bars representing the SEM of 3 independent experiments. Statistical comparisons were made to the untreated anti-LRPAP1-stained control, or as illustrated by a comparison line, by two-way ANOVA with Turkey *post-hoc* test. ns, not significant: p≥0.05, ****p<0.0001. LPS, Lipopolysaccharide; LRPAP1, Low-density lipoprotein receptor-related protein-associated protein 1.

Having found that microglia release LRPAP1, and knowing LRPAP1 is a ligand for cell surface receptors, we tested whether LRPAP1 could be found on the surface of microglia in various conditions. For this, BV-2 or CHME3 microglia were treated in serum-free media for 24 hours, then incubated with antibodies to LRPAP1 or isotype IgG, followed by fluorescently-labelled anti-Fc antibodies (the secondary antibody), and then either imaged or quantified by flow cytometry. LRPAP1 antibodies bound to the surface of all BV-2 microglia, whereas isotype control antibodies did not bind (**Figures 2A, B**). Secondary antibodies did not bind in the absence of the primary antibody (**Supplementary Figure 3**). Blocking Fc receptors had no effect on anti-LRPAP1 antibody binding (**Supplementary Figure 4**), indicating the antibody binding is not mediated by Fc receptors. Treating BV-2 cells with LPS or tunicamycin had no significant effect on the measured level of LRPAP1 on the cell surface. Similarly, anti-LRPAP1 antibodies bound to intact CHME3 microglia significantly more than control antibodies, but LPS had no effect on the apparent level of LRPAP1 on the cell surface (**Figures 2C, D**). We did not test whether tunicamycin affected levels of LRPAP1 on the cell surface of CHME3 microglia. Thus, LRPAP1 appears to be present on the surface of intact microglia, but this binding is not changed by the stress conditions used, possibly because the expression of the receptors that bind LRPAP1 have not changed in these conditions.

**Figure 3:**
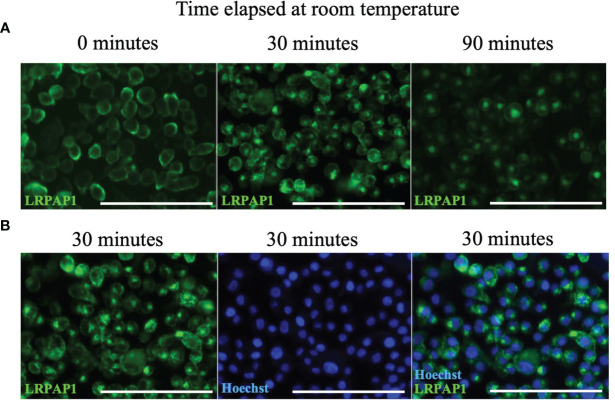
Anti-LRPAP1 antibodies are passively internalized by BV-2 microglia. BV-2 microglia were plated at 5 x 10^4^ cells/100 µL/well in serum-free medium and incubated for 24 hours. BV-2 microglia were stained with antibodies against anti-LRPAP1, followed by an Alexa Fluor 488-tagged anti-rabbit IgG secondary antibody. Cultures were kept at 4°C during the staining phase. **(A)** Representative fluorescence images detailing the timelapse of LRPAP1 internalization in BV-2 cultures moved from 4°C to room temperature. Scale = 150 µm. **(B)** Microglia that have been stained with Hoechst and anti-LRPAP1 and incubated at room temperature for 30 minutes, highlighting the peri-nuclear localization of intracellular LRPAP1. Scale = 150 µm. Images are representative of 3 independent experiments. LRPAP1, Low-density lipoprotein receptor-related protein-associated protein 1.

Antibodies binding to some cell surface receptors result in antibody internalization because dimerization of the receptor is sufficient to activate the receptor, resulting in internalization into endosome and lysosomes (37). We tested whether anti-LRPAP1 antibody binding to microglia resulted in antibody internalization, by incubating BV-2 microglia with anti-LRPAP1 antibody and fluorescently-labelled secondary antibody at 4°C [which inhibits antibody internalization (38)], and then warming the cells to room temperature for up to 90 mins, then imaging. Warming the cells induced rapid internalization of the anti-LRPAP1 antibody from the cell surface at 0 mins to a peri-nuclear compartment of the cell, presumably lysosomes, at 90 mins (**Figure 3**). We conducted this assay at room temperature, rather than at 37°C, as this meant antibody internalization was slow enough to follow over time. Thus, endogenous cell surface LRPAP1 appears to be bound to receptors, the dimerization of which results in internalization to lysosomes. Consequently, cell surface LRPAP1 might regulate microglial endocytosis or phagocytosis. Note, however, that we did not test whether antibodies to LRP1 or related receptors induce internalization.

**Figure 4:**
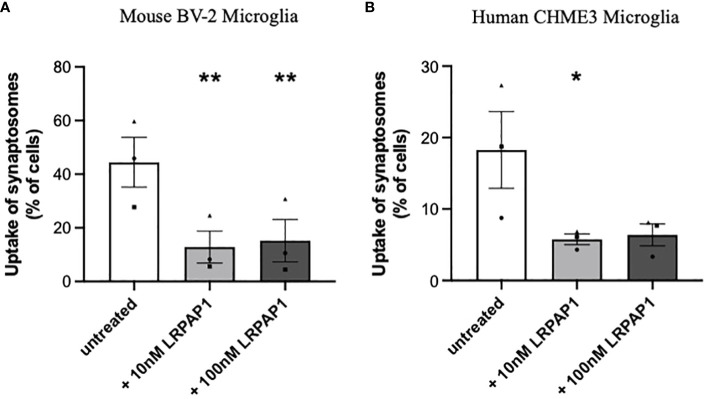
LRPAP1 inhibits the phagocytosis of synaptosomes by microglia. BV-2 microglia were plated at 2.5 x 10^4^ cells/100 µL/well. CHME3 microglia were plated at 2 x 10^4^ cells/100 µL/well. Microglia were pre-treated with or without LRPAP1 (10 or 100 nM) or cytochalasin D (10 µM) for 2 hours, then co-cultured with pHrodo-stained synaptosomes (16 µg/well) for 3 hours. **(A, B)** Phagocytosis of synaptosomes by **(A)** BV-2 microglia or **(B)** CHME3 microglia was measured by flow cytometry using the YL1-A fluorescence channel as the ‘fluorescence gate-shift’ (i.e., fluorescence data was gated at the fluorescence level of microglia treated with cytochalasin D (which inhibits phagocytosis), so that 1% of events were in the ‘fluorescence positive’ gate (i.e., engaging in phagocytosis)). Data presented as mean % of cells containing synaptic material, with error bars representing the SEM of 3 independent experiments. Statistical comparisons were made to the untreated control by one-way ANOVA with Šídák *post-hoc* test. *p<0.05, **p<0.01. LRPAP1, Low-density lipoprotein protein receptor-related protein-associated protein 1.

Having found that activated and stressed microglia release about 10 nM LRPAP1 into the extracellular medium, we wanted to test whether these levels of extracellular LRPAP1 have significant effects on microglial functions, such as the phagocytosis of synapses and cells. To do this, we isolated synapses (synaptosomes) from rat brain, labelled them with pHrodo, and measured uptake into microglia by imaging and flow cytometry. pHrodo only fluoresces significantly when in the low pH environment of lysosomes (39). Both 10 nM and 100 nM LRPAP1 significantly and substantially inhibited uptake of isolated synapses into BV-2 microglia (**Figure 4A**; **Supplementary Figure 5A**). 10 nM LRPAP1 also significantly and substantially inhibited uptake of isolated synapses into CHME3 microglia (**Figure 4B**; **Supplementary Figure 5B**). Thus, 10 nM LRPAP1 is sufficient to substantially inhibit microglial phagocytosis of synapses.

**Figure 5:**
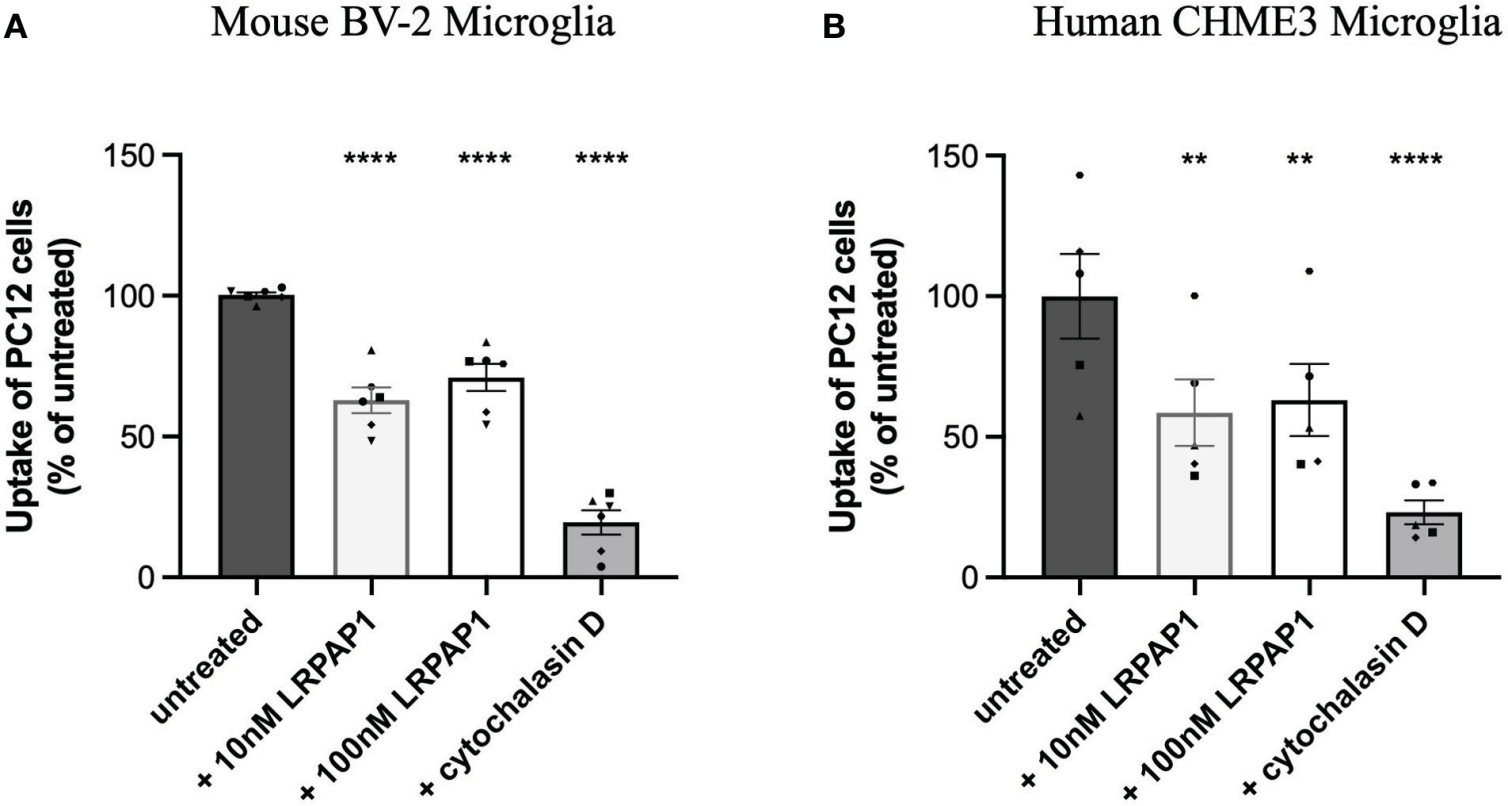
LRPAP1 inhibits PC12 phagocytosis by microglia. BV-2 microglia were plated at 2.5 x 10^4^ cells/100 µL/well. CHME3 microglia were plated at 2 x 10^4^ cells/100 µL/well. Microglia were pre-treated with or without LRPAP1 (10 or 100 nM) or cytochalasin D (10 µM) for 2 hours, then co-cultured with pHrodo-stained irradiated PC12 cells (4:1 PC12 to microglia ratio) for 2 hours. Phagocytosis of PC12 cells by microglia was measured by flow cytometry using the YL1-A fluorescence channel. **(A)** Percentage median fluorescence intensity (MFI) of treated **(A)** BV-2 and **(B)** CHME3 cultures, as a measure of PC12 uptake, normalized to the untreated microglial/PC12 co-culture control. MFI values from untreated microglial monocultures, acting as the autofluorescence control, were subtracted from all MFI values. Data is presented as percentage uptake values, with error bars representing the SEM of **(A)** 6 and **(B)** 5 independent experiments. Statistical comparisons were made to the untreated microglial/PC12 co-culture control by one-way ANOVA with Šídák *post-hoc* test. ns, p≥0.05, **p<0.01, ****p<0.0001. LRPAP1, Low-density lipoprotein protein receptor-related protein-associated protein 1.

Next, we tested whether LRPAP1 inhibited microglial phagocytosis of cells. As targets for microglial phagocytosis, we used PC12 cells (a rat neuronal-like cell line) that had been irradiated to kill them and stained with pHrodo, and then incubated with BV-2 or CHME3 microglia. Uptake of the PC12 cells into the microglia was assessed by imaging and flow cytometry. Uptake of PC12 cells into either BV-2 and CHME3 microglia was significantly inhibited by both 10 nM and 100 nM LRPAP1 (**Figures 5A, B**). Cytochalasin D, which is known to inhibit phagocytosis by disassembling microtubules (36), strongly inhibited the measured phagocytosis, confirming that uptake was by phagocytosis. Thus, 10 nM extracellular LRPAP1 is sufficient to inhibit microglial phagocytosis of cells.

**Figure 6:**
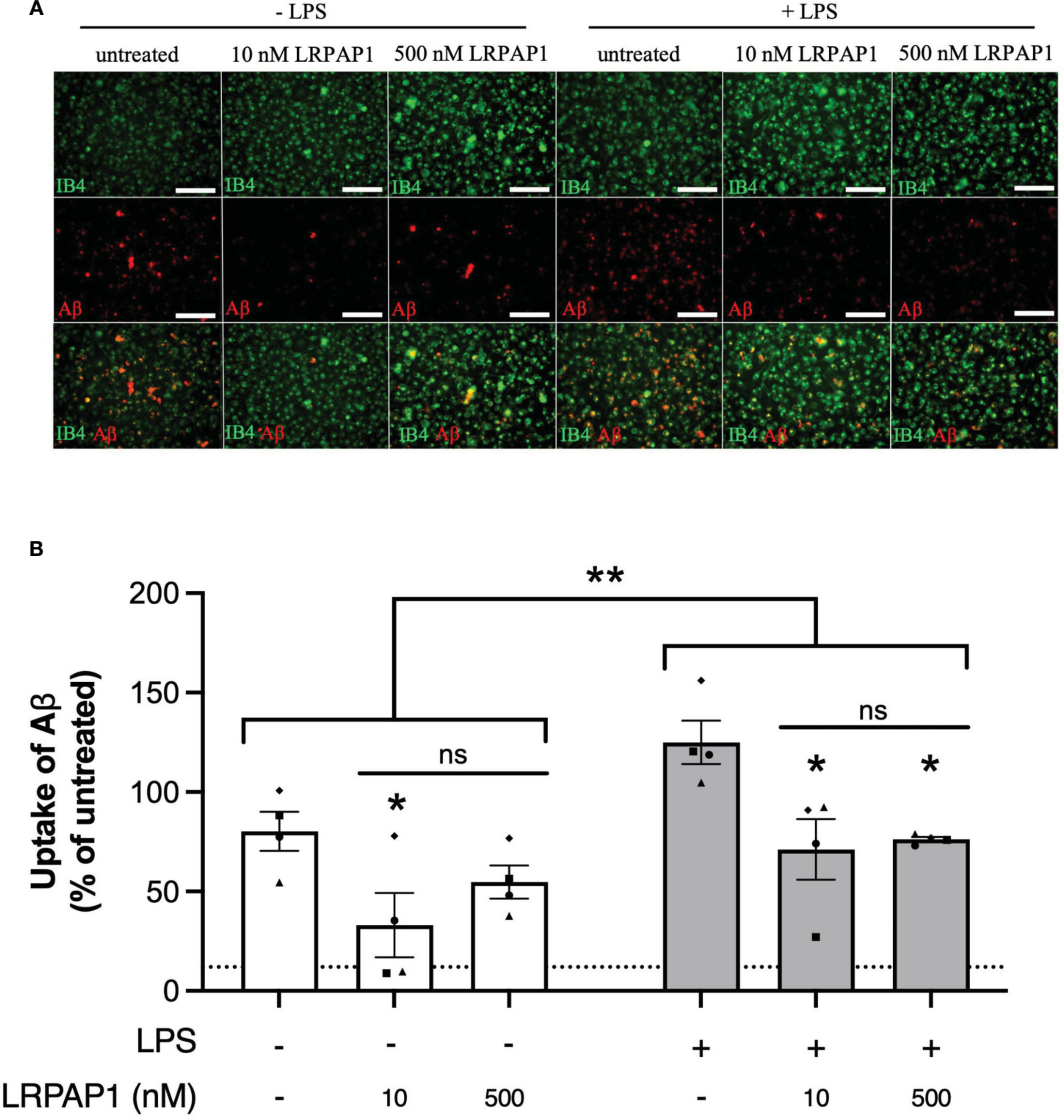
LRPAP1 inhibits Aβ uptake by mouse microglia. BV-2 microglia were treated with or without LPS (100 ng/mL) for 24 hours, then treated with HiLyte™ Fluor 555-tagged Aβ (500 nM) ± LRPAP1 (10 or 500 nM) or cytochalasin D (10 µM) for 4 hours. **(A)** Representative images of Aβ uptake by treated BV-2 microglia stained with Alexa Fluor ™ 488-tagged isolectin B4 (IB4). Scale bar = 150 µm. **(B)** Percentage median fluorescence intensity by BV-2 microglia, as a measure of Aβ uptake, was measured by flow cytometry using the YL1-A fluorescence channel and normalized to the ‘- LPS’ treated control. Untreated cultures served as the background control and were subtracted from all YL1-A values. Dotted line represents the mean percentage YL1-A value for BV-2 microglia treated with Aβ and Cytochalasin D and served as the negative control. Data presented a mean % of cells containing Aβ, with error bars representing the SEM of 4 independent experiments. For each data group (i.e. ± LPS), statistical comparisons were made to the ‘- LRPAP1 control’, or as illustrated by a comparison line, by two-way ANOVA with Turkey *post-hoc* test. ns, non significant: p≥0.05, *p<0.05, **p<0.01. LPS: Lipopolysaccharide; Aβ: Amyloid beta; LRPAP1: Low-density lipoprotein protein receptor-related protein-associated protein 1. ns, not significant.

**Figure 7:**
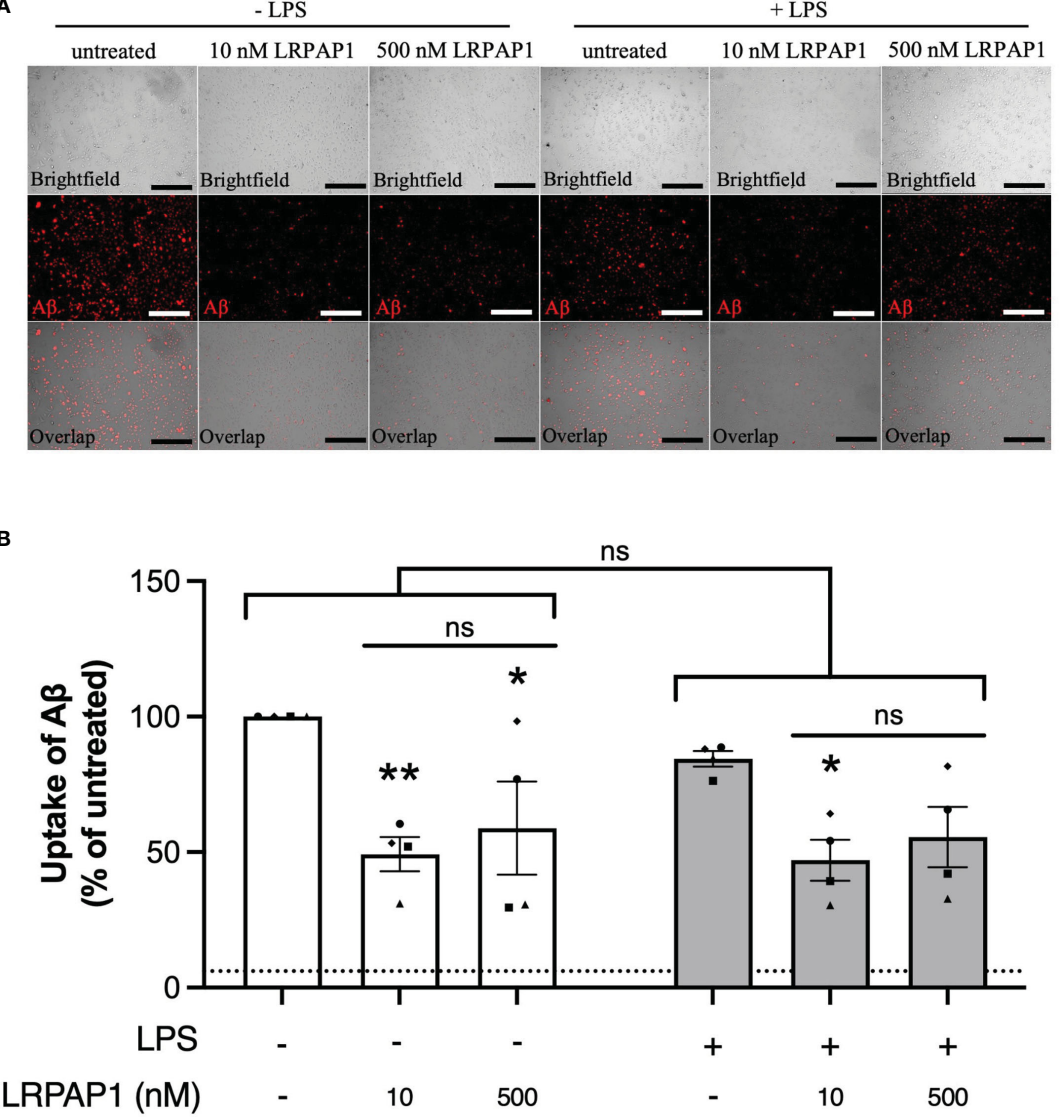
LRPAP1 inhibits Aβ uptake by human microglia. CHME3 microglia were treated with or without LPS (0.8 µg/mL) for 24 hours, then treated with HiLyte™ Fluor 555-tagged Aβ (500 nM) ± LRPAP1 (10 or 500 nM) or cytochalasin D (10µM) for 4 hours. **(A)** Representative images of Aβ uptake by treated CHME3 microglia. Scale bar = 300 µm. **(B)** Percentage median fluorescence intensity by CHME3 microglia, as a measure of Aβ uptake, was measured by flow cytometry using the YL1-A fluorescence channel and normalized to the ‘Aβ – LPS’ treated control. Untreated cultures served as the background control and were subtracted from all YL1-A values. Dotted line represents the mean percentage YL1-A value for CHME3 microglia treated with Aβ and Cytochalasin D and served as the negative control. Data presented a mean % of cells containing Aβ, with error bars representing the SEM of 4 independent experiments. For each data group (i.e. ± LPS), statistical comparisons were made to the ‘- LRPAP1 control’, or as illustrated by a comparison line, by two-way ANOVA with Turkey *post-hoc* test. ns: p≥0.05, *p<0.05, **p<0.01. LPS, Lipopolysaccharide; Aβ, Amyloid beta; LRPAP1, Low-density lipoprotein protein receptor-related protein-associated protein 1.

Having found that LRPAP1 inhibits microglial phagocytosis of synaptosomes and cells, we next tested whether LRPAP1 affected Aβ uptake by BV-2 or CHME3 microglia, an activity relevant to Alzheimer’s disease (40). We tested this using fluorescently-labelled Aβ, incubated with BV-2 or CHME3 microglia, followed by imaging and flow cytometry. Addition of 10 nM LRPAP1 significantly inhibited Aβ uptake in non-activated BV-2 and CHME3 microglia (**Figures 6**, **7**). Addition of 500 nM LRPAP1 significantly inhibited Aβ uptake in non-activated CHME3 microglia (**Figure 7**), but not BV-2 microglia (**Figure 6**). LRPAP1 does not activate microglia (**Supplementary Figure 6**), so we also tested whether LRPAP1 inhibited uptake of Aβ by microglia activated with LPS. In BV-2 microglia, both 10 and 500 nM LRPAP1 significantly inhibited Aβ uptake by activated microglia (**Figure 6**). While in CHME3 microglia, 10 and 500 nM LRPAP1 inhibited Aβ uptake by LPS-activated microglia to a similar extent, but only the inhibition by 10 nM LRPAP1 was significant (**Figure 7**). Thus, 10 nM extracellular LRPAP1 inhibits Aβ uptake by microglia, and this appears to be a saturating dose. Cytochalasin D substantially inhibited Aβ uptake indicating that most of the uptake was by phagocytosis or endocytosis (**Figures 6**, **7**; **Supplementary Figure 7**).

**Figure 8:**
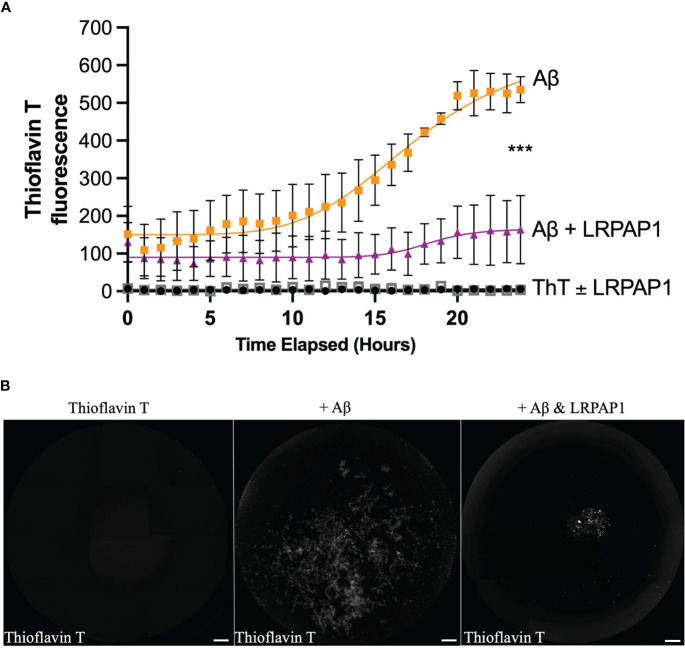
LRPAP1 inhibits fibrillization of Aβ. Thioflavin T (10 µM), on its own or prepared with Aβ (5 µM) ± LRPAP1 (500 nM), was incubated at 31.5°C with periodic orbital shaking (lasting 10 seconds) every 10 minutes for 24 hours. Every hour, after orbital shaking, thioflavin T fluorescence was measured. **(A)** Timelapse of thioflavin T fluorescence. **(B)** Representative images of thioflavin T fluorescence after 24 hours. Note that thioflavin T fluoresces when bound to fibrillized Aβ. Scale = 500 µm. Error bars represent the SEM of 3 independent experiments. Statistical comparisons between ‘Aβ’ and ‘Aβ + LRPAP1’ was made by one-way ANOVA with Šídák *post-hoc* test. ns: p≥0.05, ***p<0.001. Aβ, Amyloid beta; LRPAP1, Low-density protein receptor associated protein 1.

As LRPAP1 is a chaperone, and chaperones often work by non-specifically binding exposed hydrophobic portions of proteins to prevent aggregation (41), we wondered whether LRPAP1 might affect Aβ aggregation. To test this, we examined the kinetics of aggregation of 5 µM Aβ in the presence of thioflavin T (which fluoresces when bound to β sheet) ± 500 nM LRPAP1 using a fluorimeter under standard conditions known to promote fibrillization (42, 43). In the absence of LRPAP1, Aβ aggregated with standard sigmoidal kinetics (**Figure 8A**). In the presence of LRPAP1, Aβ aggregation was significantly and substantially inhibited. Imaging of the wells at the end of the 24-hour incubation revealed standard, Thioflavin T-positive Aβ fibrils in the absence of LRPAP1 (**Figure 8B**). In the presence of LRPAP1, there were very few Aβ fibrils, but there was a small amount of Thioflavin T-positive Aβ in amorphous aggregates in the center of the wells (**Figure 8B**). Thus, LRPAP1 appears to strongly inhibit Aβ aggregation.

## 4 Discussion

We found here that BV-2 and CHME3 microglia released nanomolar levels of LRPAP1 into the medium, when inflammatory activated with LPS or ER stressed with tunicamycin. Addition of these nanomolar levels of LRPAP1 to the medium significantly inhibited microglial phagocytosis of synapses, cells, and Aβ. LRPAP1 also inhibited Aβ aggregation. Thus, LRPAP1 release from microglia potentially regulates microglial phagocytosis and Aβ pathology.

We did not investigate the mechanism by which microglia released LRPAP1, when untreated or in response to LPS and tunicamycin, except to confirm that it was not due to necrosis. One potential route to the cell surface is via the normal secretory pathway (ER ➔ Golgi ➔ cell surface) attached to LRP1 or other receptors of the LDL receptor family (44). Once at the cell surface, LRPAP1 will dissociate from these receptors, depending on the dissociation constants for these receptors and the concentration of free LRPAP1 in the extracellular medium. LPS and tunicamycin might increase the flux of LRPAP1 and these receptors through the secretory pathway, resulting in increased LRPAP1 release, or alternatively promote release of LRPAP1 independent of LRP1 and related receptors. We recently reported that LPS can induce microglia to release another ER-resident chaperone, calreticulin (30), and others have shown that ER stress can induce the release of calreticulin and other ER-resident chaperones (30, 45).

We found LRPAP1 on the surface of live microglia, as measured by binding of anti-LRPAP1 antibodies. We did not investigate what this LRPAP1 was bound to on the cell surface, but clearly this might be LRP1 or other receptors of the LDL receptor family. Thus, the finding of LRPAP1 on the surface of live microglia suggests the possibility that extracellular LRPAP1 may be bound to one or more of these receptors, and thereby regulating these receptors. It has previously been found that endogenous LRPAP1 is found bound to LRP1 on the surface of live cells, and blocks the binding of extracellular ligands of LRP1 (44). We found that the measured cell surface LRPAP1 did not change with LPS or tunicamycin, which may mean that the expression of the receptors that bind LRPAP1 on the cell surface does not change under these conditions.

Anti-LRPAP1 antibodies bound to the surface of live microglia, and at temperatures above 4°C, were internalized into a peri-nuclear compartment, probably lysosomes. A possible interpretation of this is that antibody-induced crosslinking of LRPAP1 bound to LRP1 or related receptors, induces dimerization of these receptors, which in turn induces endocytosis of these receptors with bound LRPAP1 and antibodies. However, we did not investigate the mechanism of this uptake.

LRP1 has four potential ligand-binding domains: domain I does not bind any known ligands, domains II and IV binds all known ligands (46), and domain III binds ApoE and blood coagulation factor VIII (47, 48), while LRPAP1 can bind domains II, III and IV in competition with all known ligands (46–50). Thus, extracellular LRPAP1 potentially regulates the affinity of all physiological ligands for LRP1. Physiological ligands may activate LRP1 and related receptors by binding two or more receptor molecules, resulting in dimerization of the intracellular domain, leading to phosphorylation of tyrosine residues in these domains, which in turn recruit other signaling or scaffolding proteins (51). LRPAP1 may inhibit simply by binding to the ligand binding sites as a monomer, thereby preventing dimerization, although other potential modes of inhibition are possible.

LRP1 and related receptors of the LDL receptor family are known to mediate endocytosis or phagocytosis of a wide range of extracellular targets, including Aβ, TAU, ApoE and apoptotic cells, via ligand binding to these receptors (6, 28, 52). The dissociation constant (K_D_) of LRPAP1 for these receptors, reported in the literature, are as follows: LRP1 [1-14 nM (9, 14)], LRP-2 [K_D_: 8 nM (11)], ApoER2 [K_D_: 5 nM (12)], VLDL receptor [K_D_: 0.7 nM (10)] and LDL receptor [K_D_: 250 nM (13)]. Thus, if microglia can release LRPAP1 up to an extracellular concentration of 10 nM, as we found, then we might expect 10 nM extracellular LRPAP1 to inhibit endocytosis or phagocytosis mediated by LRP1, LRP2, ApoER2 and VLDLR. We confirmed that 10 nM extracellular LRPAP1 significantly inhibited microglial uptake of Aβ, isolated synapses (synaptosomes) and cells (dead PC12 cells). We did not investigate which receptors mediated this uptake or inhibition, but clearly LRP1 could be involved. Microglia have been shown to express LRP1, ApoER2, VLDLR and LDLR (53), but it is not known whether they express LRP2. Microglial phagocytosis of synapses is important in brain development, and disruption of this phagocytosis may lead to developmental pathologies, such as autism (54). Microglial phagocytosis of synapses may contribute to synapse loss in neurogenerative diseases, such as Alzheimer’s disease (55), and, if so, we might expect extracellular LRPAP1 to be protective. Microglial phagocytosis of neurons may contribute to brain development (56–58), but also neurodegeneration (59), where again we might expect LRPAP1 to be protective by preventing excessive phagocytosis, but this would require testing.

Microglial uptake of Aβ is known to be partly mediated by LRP1 (22), and we found that 10 nM LRPAP1 inhibited Aβ uptake. Thus, extracellular LRPAP1 is likely to be detrimental in Aβ pathology by blocking clearance of extracellular Aβ. However, microglial uptake of Aβ can also inflammatory activate microglia, which can potentially be detrimental. We have previously shown that extracellular LRPAP1 can prevent neuronal loss induced by LPS or Aβ (60). Thus, the LRPAP1 released by activated microglia is potentially neuroprotective. Note that the inhibition of Aβ uptake could potentially be due to LRPAP1 binding Aβ (see below), rather than LRPAP1 directly inhibiting LPR1.

LRP1, expressed by the blood-brain barrier, is known to mediate transport of Aβ out of the brain (61), and extracellular LRPAP1 is known to inhibit this (62). Thus, if extracellular LRPAP1 levels increase, potentially as a result of inflammation, this might inhibit Aβ export from the brain, potentially increasing Aβ levels within the brain.

LRP1 is known to bind and mediate uptake of oligomeric TAU into cells, and this is fully blocked by LRPAP1 with an inhibition constant (K_i_) of 5 nM extracellular LRPAP1 (52); and TAU spreading in mouse brain is mainly mediated by LRP1 (52). Thus, extracellular LRPAP1 can potentially block TAU spreading in Alzheimer’s disease and other TAU pathologies.

LRP1 can also mediate microglial phagocytosis of myelin, which is inhibited by extracellular LRPAP1 (63), albeit LRPAP1 was added at supraphysiological levels. Thus, if LRPAP1 is released during demyelination it might inhibit myelin clearance, which is thought to be important in multiple sclerosis (63).

LRPAP1 is known to bind Aβ (64), and we found that LRPAP1 inhibited Aβ aggregation into fibrils. We did not investigate the mechanism of this inhibition, but as LRPAP1 is an ER chaperone, it may simply bind to any hydrophobic patches exposed on Aβ (such as any beta sheet) aiding refolding or preventing aggregation via these patches (8). If so, then LRPAP1 may be an extracellular chaperone, as well as an intracellular chaperone.

For these aggregation studies, we used 5 µM Aβ, because this is the minimum concentration to be able to detect aggregation *in vitro*. To block aggregation in this assay we used 500 nM LRPAP1, which is a 1:10 molar ratio to Aβ, but 100-fold higher concentration than that released by microglia. Thus, it would be important to test whether LRPAP1 could inhibition Aβ aggregation at more physiological concentrations and conditions, both *in vitro* and *in vivo*. If extracellular LRPAP1 does indeed inhibit Aβ aggregation in the brain, it would be interesting to investigate whether a fall in LRPAP1 precipitates Aβ aggregation in human brain, or whether LRPAP1 could be used therapeutically to prevent or treat Aβ pathology.

Although the released amount of LRPAP1 under stress/inflammation was around 10 nM, this was measured for particular cells in particular conditions at a particular density of cells. So, the concentration of released LRPAP1 might be different in different conditions. Thus, it is informative to have some idea of the concentration dependence of the effects of LRPAP1 on cellular functions, which we assessed using high and low nanomolar LRPAP1, in part to know over what range of concentrations LRPAP1 is affecting cellular functions.

The concentration of LRPAP1 normally found by mass spectrometry in human blood plasma is about 800 ng/l, equivalent to about 20 pM (*PeptideAtlas*), and plasma levels are negatively correlated with cognition in some conditions (65). LRPAP1 is also present in the cerebrospinal fluid, and mildly decreases with minor cognitive impairment and mildly increases in Alzheimer’s disease (66). The concentration of LRPAP1 in brain extracellular space is unknown. Our measurement of LRPAP1 release was in BV-2 and CHME3 microglial cell lines, which may differ significantly from microglia *in vivo*, so it would be useful to expand these measurements to primary cells, other cell types and the brain *in vivo*. Microglia in the brain are normally less activated than in culture, and basal stress or activation in culture may potentially cause some basal level of LRPAP1 release. So, we cannot rule out that the basal release of LRPAP1 that we found in culture was induced by the serum-free conditions in which release was measured. However, microglia can be become stressed or inflammatory activated *in vivo*, and any potential LRPAP1 release would be into a smaller extracellular space (than in culture), so potentially resulting in higher extracellular concentrations. Nevertheless, this is speculation prior to actually measuring LRPAP1 levels in the extracellular space of the brain, and determining whether any such LRPAP1 actually regulates LRP1 and related receptors.

The research reported here mirrors our previous finding that inflamed and stressed microglia release the ER chaperone calreticulin, which inhibits Aβ aggregation and affects microglial functions (30). We do not know whether ER chaperones have a common mechanism of release in these conditions, or whether they have a common mechanism of inhibiting Aβ aggregation. The mechanisms by which calreticulin and LRPAP1 affect microglial function is probably different, in that calreticulin appears to activate microglia via TLR4 (30), while LRPAP1 probably inhibits microglial phagocytosis via LRP1 and related receptors of the LDL receptor family. Calreticulin and LRPAP1 both bind LRP1, but calreticulin is an agonist and co-receptor with LRP1 (67, 68), while LRPAP1 is an antagonist of LRP1. LRPAP1 release from stressed microglia and cells generally has wider implications than the release of calreticulin, because it inhibits a range of receptors that have many different cellular functions.

Outside the brain, LRP1 and related LDL family receptors regulate a wide range of cellular processes, including endocytosis, phagocytosis, chemotaxis and signaling in response to dozens of ligands (1–4). All of these are potentially regulated by extracellular LRPAP1, but clearly this requires further investigation.

## Data availability statement

The original contributions presented in the study are included in the article/**Supplementary Material**. Further inquiries can be directed to the corresponding author.

## Author contributions

KR: Formal Analysis, Investigation, Methodology, Validation, Visualization, Writing – original draft, Writing – review & editing. GB: Conceptualization, Supervision, Writing – original draft, Writing – review & editing.
