# Supplementary material for: LRPAP1 is released from activated microglia and inhibits microglial phagocytosis and amyloid beta aggregation
Source: Front Immunol. 2023 Nov 16;14:1286474. doi: 10.3389/fimmu.2023.1286474 (PMC10687467; doi:10.3389/fimmu.2023.1286474)
Supplement: Supplementary file 1 [file DataSheet_1.pdf]

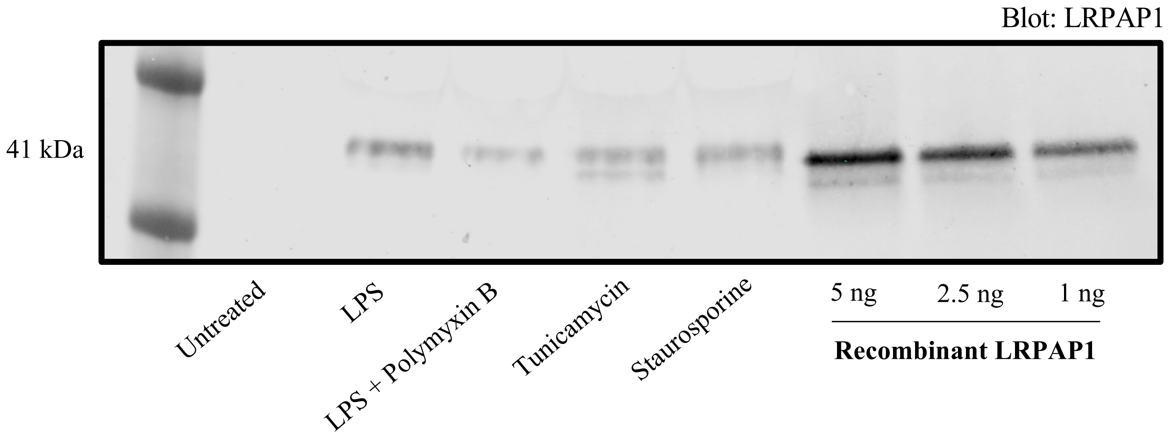

Supplementary Figure 1. Western blot against LRPAP1 with recombinant LRPAP1 standard curve. BV-2 microglia were treated with LPS (100 ng/mL) ± polymyxin B (10 U/mL), tunicamycin (2 μg/mL), or staurosporine (1000 nM) for 24 hours. Supernatants were removed and assessed for LRPAP1 release by quantitative densitometry of western blots using internal three-point standard curves of recombinant LRPAP1. Image depicts a representative western blot image of LRPAP1 release from treated BV-2 microglia, with an example standard curve of recombinant LRPAP1. LRPAP1: Low-density lipoprotein protein receptor-related protein-associated protein 1.

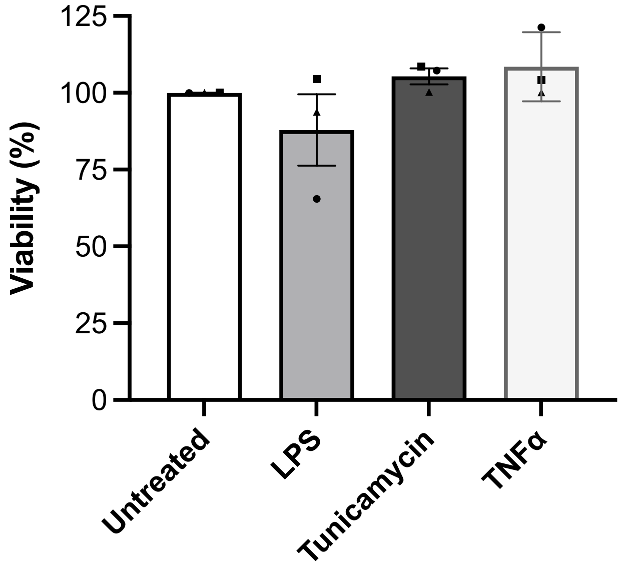

Supplementary Figure 2. Viability of stressed CHME3 microglia. CHME3 microglia were treated with or without LPS (0.8 µg/mL), tunicamycin (2 µg/mL), or TNF-α (50 ng/mL) for 24 hours. Percentage viability of CHME3 stained with propidium iodide and Hoechst, normalized to the untreated control. CHME3 viability data is represented as error bars indicating the SEM of 3 independent experiments. LPS: Lipopolysaccharide; TNF-α: Tumour necrosis factor α.

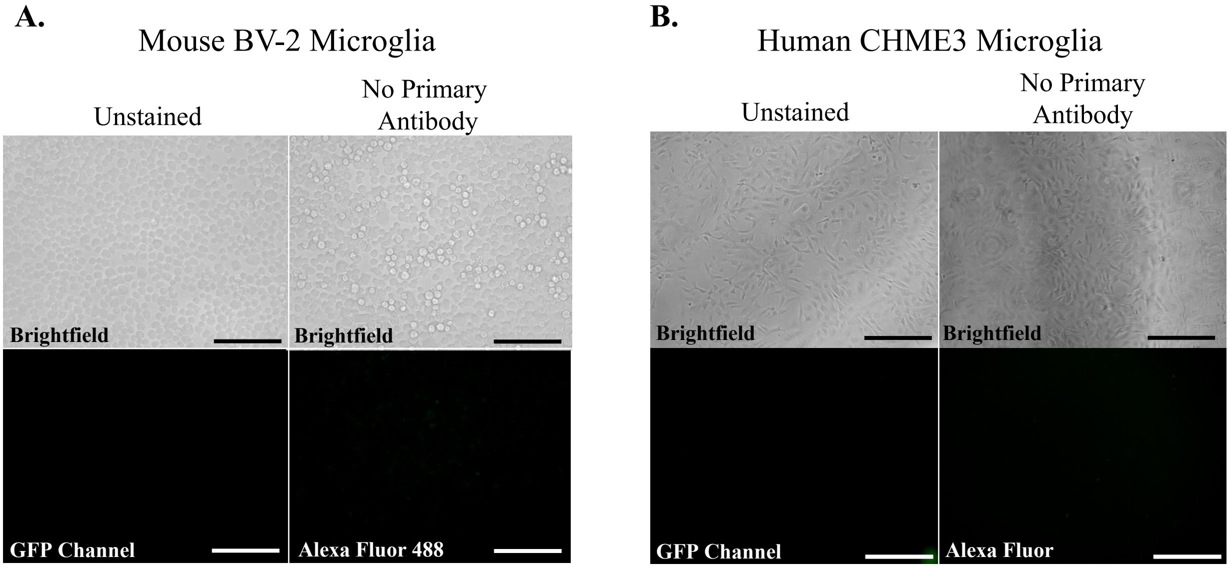

Supplementary Figure 3. Microglia do not autofluoresce in the GFP channel, and do not non-specifically bind secondary antibodies to anti-rabbit IgG. BV-2 microglia were plated at 5 x 10^4 cells/ 100μL/ well in serum-free medium and treated for 24 hours. CHME3 microglia were plated at 1.5 x 10^4 cells/ 100μL/ well in serum-free medium for 24 hours. (**A**) Representative fluorescence images of (**A**) BV-2 microglia (Scale = 150 μm) or (**B**) CHME3 microglia (Scale = 300 μm) that have not been stained (i.e., unstained control), or have only been stained with an Alexa Fluor 488-tagged anti-rabbit IgG secondary antibody (i.e., secondary control). Cultures were kept at 4°C during the staining phase. Data is representative of 3 independent experiments.

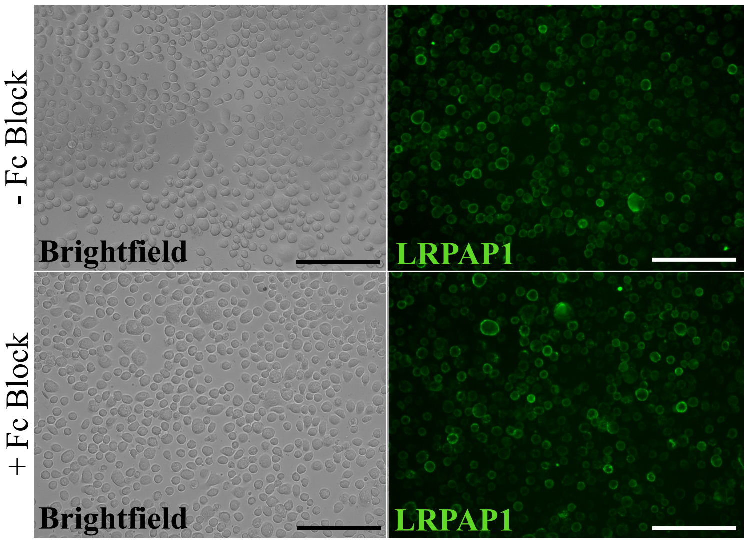

Supplementary Figure 4. Blocking mouse Fc receptors does not alter the surface staining of anti-LRPAP1 antibodies. BV-2 microglia were plated at 5 x 10^4 cells/ 100µL/ well in serum-free medium and incubated for 24 hours. BV-2 microglia were pre-treating with mouse seroblock FcR BUF041A (5 µg/mL) for 20 minutes before staining with antibodies against anti-LRPAP1, followed by an Alexa Fluor 488-tagged anti-rabbit IgG secondary antibody. Cultures were kept at 4ºC during the staining phase. Scale = 150 µm. Data is representative of 3 independent experiments. LRPAP1: Low-density lipoprotein receptor-related protein-associated protein 1.

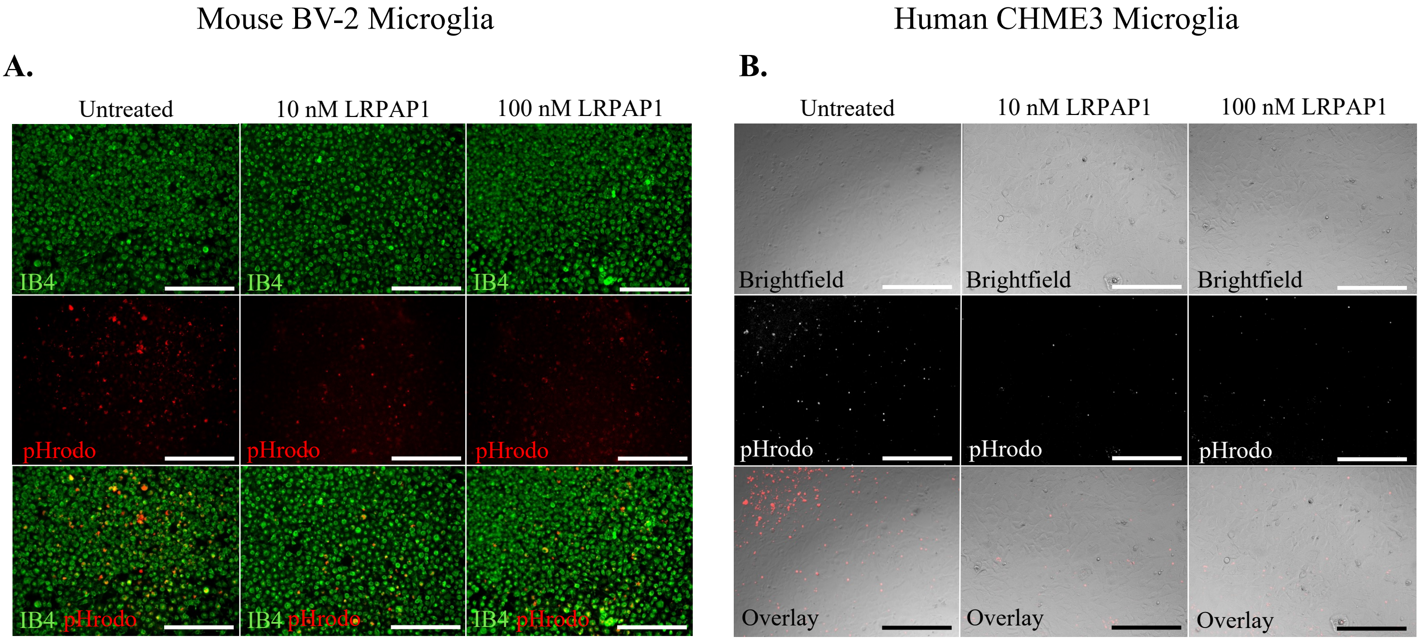

Supplementary Figure 5. LRPAP1 inhibits the phagocytosis of synaptosomes by microglia. BV-2 microglia were plated at 2.5 x 10^4 cells/ 100 µL/ well. CHME3 microglia were plated at 2 x 10^4 cells/ 100 µL/ well. Microglia were pre-treated with or without LRPAP1 (10 or 100 nM) or cytochalasin D (10 µM) for 2 hours, then co-cultured with pHrodo-stained synaptosomes (16 µg /well) for 3 hours. (**A**) Representative images of pHrodo-labelled synaptosome uptake by treated BV-2 microglia labelled with Alexa Fluor 488-conjugated isolectin B4 (IB4). Scale = 300 µm. (**B**) Representative images of pHrodo-labelled synaptosome uptake by treated CHME3 microglia. Scale = 400 µm Data is representative of 3 independent experiments. LRPAP1: Low-density lipoprotein receptor-related protein-associated protein 1.

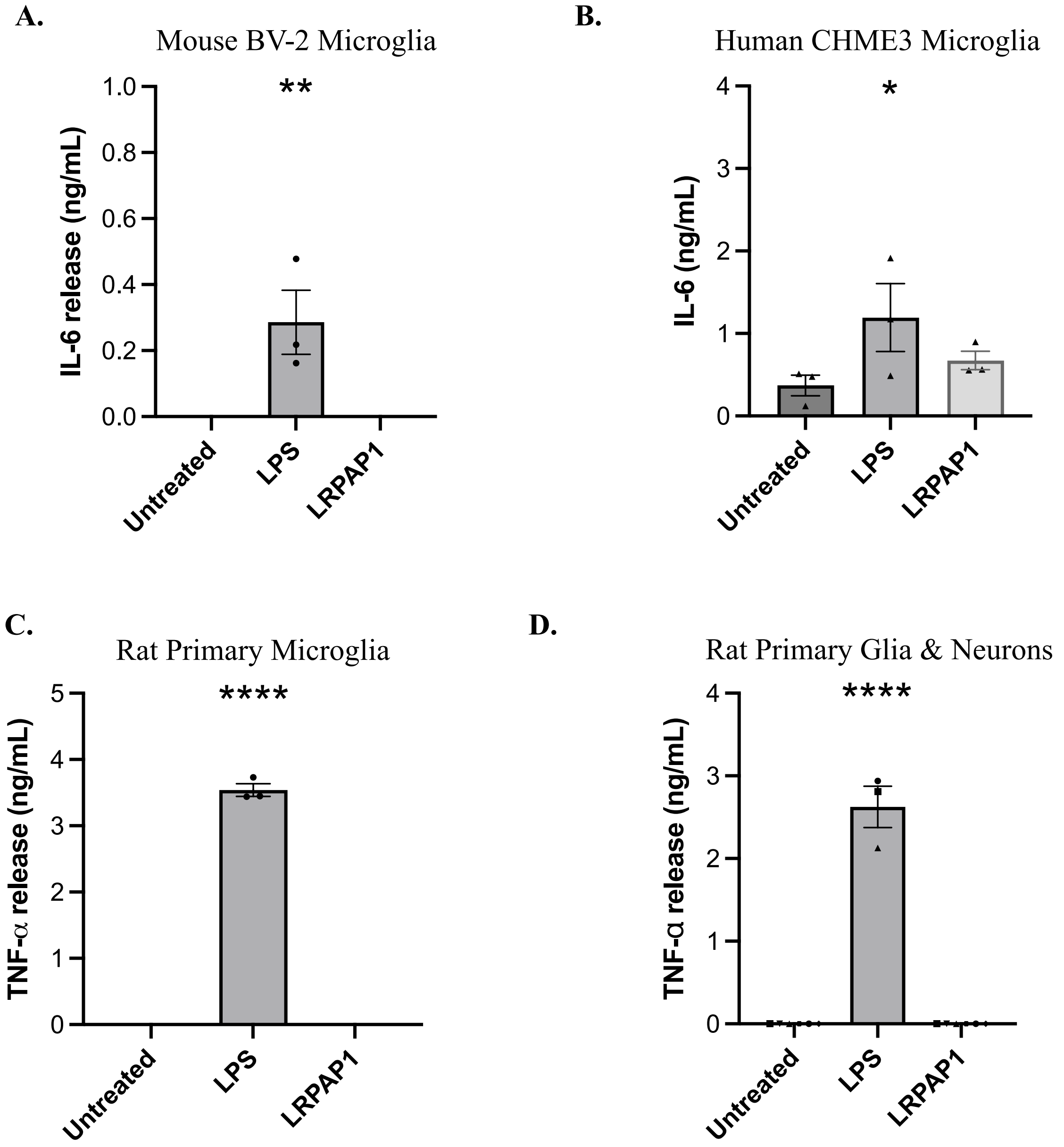

Supplementary Figure 6. **LRPAP1 does not stimulate the release of pro-inflammatory cytokines from microglia.** Cytokine release was quantified from the supernatants of treated microglia by ELISA. (**A**) IL-6 in culture media of BV-2 microglia, 20 hours after treatment with 100 ng/mL LPS or 100 nM LRPAP1. (**B**) IL-6 in culture media of CHME3 microglia, 20 hours after treatment with 0.8 µg/mL LPS or 200 nM LRPAP1. (**C**) TNF-α in culture media of rat primary microglia, 20 hours after treatment with 100 ng/mL LPS or 100 nM LRPAP1. (**D**) TNF-α in culture media of rat primary glia-neuronal mixed cultures, 3 days after treatment with 100 ng/mL LPS or 100 nM LRPAP1. Data presented as mean and SEM of at least 3 independent experiments. Statistical comparisons were made to the untreated control by one-way ANOVA. *: $p<0.05$, **: $p<0.01$, ****: $P<0.0001$. IL-6: Interleukin 6; TNF-α: Tumour necrosis factor alpha; LPS: Lipopolysaccharide; ELISA: Enzyme-linked immunosorbent assay; LRPAP1: Low-density lipoprotein receptor-related protein-associated protein 1

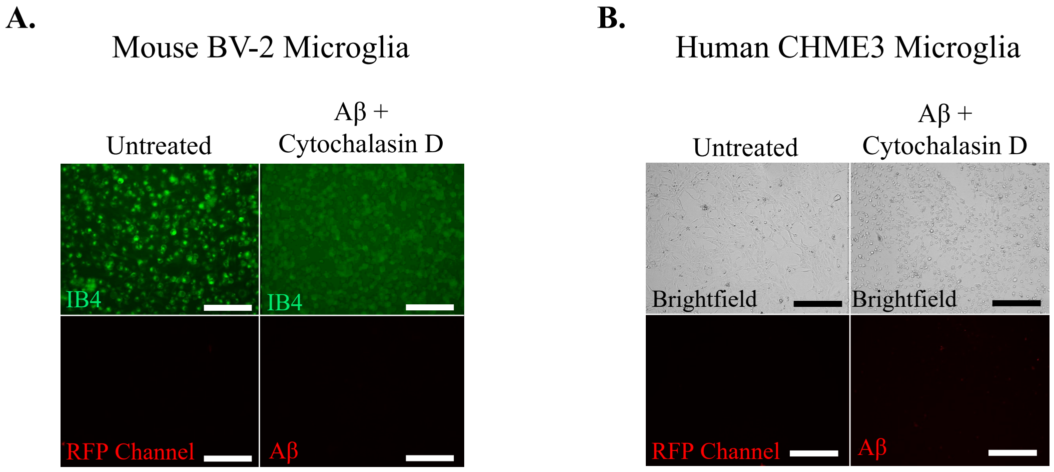

Supplementary Figure 7. Microglia do not autofluoresce in the RFP channel and cannot uptake Aβ when endocytic mechanisms are inhibited. BV-2 microglia or CHME3 microglia were treated with HiLyte™ Fluor 555-tagged Aβ (500 nM) ± cytochalasin D (10µM) for 4 hours. (**A-B**) Representative fluorescence images of (**A**) BV-2 microglia (Scale = 150 µm) or (**B**) CHME3 microglia (Scale = 300 µm) that have not been treated (i.e., untreated control), or have been treated with the endocytic inhibitor Cytochalasin D (i.e., negative control). Data is representative of 3 independent experiments.
